# Künstliche Intelligenz und sichere Gesundheitsdatennutzung im Projekt KI-FDZ: Anonymisierung, Synthetisierung und sichere Verarbeitung für Real-World-Daten

**DOI:** 10.1007/s00103-023-03823-z

**Published:** 2024-01-04

**Authors:** Fabian Prasser, Nico Riedel, Steven Wolter, Dörte Corr, Marion Ludwig

**Affiliations:** 1 Center für Health Data Science, Berlin Institute of Health der Charité – Universitätsmedizin Berlin, Charitéplatz 1, 10117 Berlin, Deutschland; 2 Forschungsdatenzentrum Gesundheit, Bundesinstitut für Arzneimittel und Medizinprodukte (BfArM), Bonn, Deutschland; 3 Fraunhofer-Institut für Digitale Medizin MEVIS, Bremen, Deutschland; 4 InGef – Institut für angewandte Gesundheitsforschung Berlin GmbH, Berlin, Deutschland

**Keywords:** Big data, Datenaustausch, Medizinische Forschung, Datenschutztechnologien, Machine Learning, Big data, Data exchange, Medical research, Data protection technologies, Machine learning

## Abstract

Die zunehmende Digitalisierung des Gesundheitswesens ist verbunden mit einem stetig wachsenden Datenvolumen, das durch Sekundärnutzung wertvolle Erkenntnisse über Diagnostik, Behandlungsprozesse und die Versorgungsqualität liefern kann. Das Forschungsdatenzentrum Gesundheit (FDZ) soll hierfür eine Infrastruktur bereitstellen. Dabei sind sowohl der Schutz der Privatsphäre der Patientinnen und Patienten als auch optimale Auswertungsmöglichkeiten von zentraler Bedeutung. Künstliche Intelligenz (KI) bietet hierfür ein doppeltes Potenzial. Zum einen ermöglichen Methoden des Machine Learning die Verarbeitung großer Datenmengen und die Analyse komplexer Zusammenhänge. Zum anderen können mithilfe von KI erzeugte synthetische – also künstliche – Daten die Privatsphäre schützen.

In diesem Beitrag wird das Projekt KI-FDZ vorgestellt, welches innovative Technologien erforscht, die eine sichere Bereitstellung von Sekundärdaten für Forschungszwecke gewährleisten können. Es wird ein mehrschichtiger Ansatz untersucht, bei dem Maßnahmen auf Datenebene auf unterschiedliche Weise mit der Verarbeitung in sicheren Umgebungen kombiniert werden können. Dazu werden unter anderem Anonymisierungs- und Synthetisierungsmethoden anhand von 2 konkreten Anwendungsbeispielen evaluiert. Zudem wird untersucht, wie das Erstellen von Pipelines für maschinelles Lernen und die Ausführung von KI-Algorithmen in sicheren Umgebungen gestaltet werden können. Vorläufige Ergebnisse deuten darauf hin, dass mit diesem Ansatz ein hohes Maß an Schutz bei gleichzeitig hoher Datenvalidität erreicht werden kann. Der im Projekt untersuchte Ansatz kann ein wichtiger Baustein für die sichere Sekundärnutzung von Gesundheitsdaten sein.

## Abstract (en)

The increasing digitization of the healthcare system is leading to a growing volume of health data. Leveraging this data beyond its initial collection purpose for secondary use can provide valuable insights into diagnostics, treatment processes, and the quality of care. The Health Data Lab (HDL) will provide infrastructure for this purpose. Both the protection of patient privacy and optimal analytical capabilities are of central importance in this context, and artificial intelligence (AI) provides two opportunities. First, it enables the analysis of large volumes of data with flexible models, which means that hidden correlations and patterns can be discovered. Second, synthetic – that is, artificial – data generated by AI can protect privacy.

This paper describes the KI-FDZ project, which aims to investigate innovative technologies that can support the secure provision of health data for secondary research purposes. A multi-layered approach is investigated in which data-level measures can be combined in different ways with processing in secure environments. To this end, anonymization and synthetization methods, among others, are evaluated based on two concrete application examples. Moreover, it is examined how the creation of machine learning pipelines and the execution of AI algorithms can be supported in secure processing environments. Preliminary results indicate that this approach can achieve a high level of protection while maintaining data validity. The approach investigated in the project can be an important building block in the secure secondary use of health data.

## Hintergrund

### Sekundärnutzung von Gesundheitsdaten

Die fortschreitende Digitalisierung des Gesundheitswesens generiert ein steigendes Volumen an Daten. Konsequenterweise gewinnt auch Forschung, die diese Gesundheitsdaten nutzt – obwohl sie ursprünglich nicht für Forschungszwecke gesammelt wurden –, zunehmend an Bedeutung [1]. Sekundär nutzbare Gesundheitsdaten, beispielsweise aus der Routinedokumentation oder von Krankenkassen, können wertvolle Informationen über Diagnostik, Behandlungen und Outcomes sowie Krankheitsverläufe, Kostenstrukturen und die Versorgungsqualität liefern. Werden bestehende Limitationen berücksichtigt, können Sekundärdaten eine wertvolle Quelle für die Forschung in verschiedenen Bereichen, wie der personalisierten Medizin, der Versorgungsforschung und Public Health, sein [2, 3]. Neben der Beantwortung von Forschungsfragestellungen lassen sich auch Trends und Muster identifizieren, die für die Planung und Steuerung des Gesundheitssystems durch politische Entscheidungsträger relevant sind.

### Das Forschungsdatenzentrum Gesundheit

Das am Bundesinstitut für Arzneimittel und Medizinprodukte (BfArM) im Aufbau befindliche Forschungsdatenzentrum Gesundheit (FDZ) soll als wichtiger Baustein des deutschen Ökosystems für die Sekundärnutzung von Gesundheitsdaten, basierend auf den §§ 303a bis 303f Sozialgesetzbuch V sowie der Datentransparenzverordnung, in einem ersten Schritt die Abrechnungsdaten der Gesetzlichen Krankenversicherungen (GKV) für definierte Forschungszwecke zugänglich machen [4]. Damit baut das BfArM eine Basisinfrastruktur auf, die perspektivisch für die Vernetzung verteilter Primärforschungssysteme und die Moderation von Forschungsanfragen dienen soll. So verspricht insbesondere die Möglichkeit von Datenspenden durch Nutzerinnen und Nutzer der elektronischen Patientenakte (ePA) die Granularität der Forschungsdaten, die über das FDZ angefragt werden können, deutlich zu erhöhen. Um dies erfolgreich umsetzen zu können, sind Maßnahmen zur Etablierung einheitlicher Standards, der Förderung von Interoperabilität und zum Aufbau einer sicheren Infrastruktur mit datenschutzkonformen Zugangsmechanismen erforderlich.

### Datenschutz als Herausforderung

Die Sekundärnutzung von Gesundheitsdaten für die Forschung unterliegt in Deutschland strengen gesetzlichen Vorgaben, um den Schutz der Privatsphäre der Patientinnen und Patienten zu gewährleisten. Zentrale Regelungen finden sich im Bundesdatenschutzgesetz [5] und in der europäischen Datenschutzgrundverordnung (DSGVO; [6]). Insbesondere muss sichergestellt sein, dass die Identifikation einzelner Versicherter ausgeschlossen ist. Daneben sollte zur Wahrung von Unternehmensgeheimnissen auch ein Rückschluss auf einzelne Gesundheitsdienstleister ausgeschlossen sein. Neben einer Informationssicherheitsstrategie nach dem neuesten Stand der Technik zur Sicherstellung der Vertraulichkeit, Integrität und Verfügbarkeit der Systeme und Daten erfordert dies insbesondere auch den Einsatz moderner Datenschutzverfahren. Dazu gehören auch Anonymisierungsverfahren auf Datenebene, die eine Reidentifizierung der Gesundheitsdaten stark erschweren bzw. unmöglich machen. Dabei ist aber zu beachten, dass eine Reduktion von Reidentifizierungspotenzialen immer auch eine Reduktion der Validität und damit Nützlichkeit der Daten nach sich zieht und beide Aspekte gegeneinander abgewogen werden müssen. Insbesondere klassische Anonymisierungsverfahren, die auf einer Verrauschung oder Vergröberung von Daten basieren, kommen in Bezug auf die Validität der geschützten Daten zunehmend an ihre Grenzen [7]. Moderne Methoden für den Aufbau sicherer Verarbeitungsumgebungen oder die KI-basierte Anonymisierung durch Synthetisierung haben ein großes Potenzial, diese Abwägungen zu verbessern, wie im Folgenden erläutert wird.

### Die Rolle künstlicher Intelligenz

Methoden der sogenannten künstlichen Intelligenz (KI) wird in vielen Bereichen ein disruptives Potenzial zugesprochen. Die Analyse von Gesundheitsdaten mittels KI wird perspektivisch zu weitreichenden Veränderungen in der Gesundheitsversorgung führen. KI-Verfahren erlauben es, große Mengen an Gesundheitsdaten ohne möglichen Bias durch explizite Modellierung zu verarbeiten, Datenmuster und Zusammenhänge zu erkennen sowie präzise Vorhersagen zu treffen. Entsprechend können komplexe medizinische Daten aus Behandlungsakten, bildgebenden Verfahren, genetischen Informationen und klinischen Studien mithilfe von KI analysiert werden, um Diagnosen zu verbessern, personalisierte Behandlungspläne zu entwickeln und die Gesundheitsversorgung zu optimieren [8]. Ein wichtiger Anwendungsfall ist die Entwicklung von personalisierten Behandlungen, Gesundheitsdiensten und maßgeschneiderten Empfehlungen für Patientinnen und Patienten, um ihre Gesundheit aktiv zu fördern und Krankheiten vorzubeugen. Dazu kann beispielsweise gehören, Risikofaktoren zu identifizieren und Bürgerinnen und Bürgern gezielt spezifische Screening- oder Präventionsmaßnahmen anzubieten.

Gleichzeitig können KI-Methoden auch dazu beitragen, Gesundheitsdaten bei der Sekundärnutzung besser zu schützen. Eine wichtige Methode ist dabei die Datensynthetisierung als Anonymisierungsverfahren. Dabei approximieren maschinelle Lernalgorithmen (Machine Learning und generative Modelle als Formen von KI) die Verteilungen und Struktur von Originaldatensätzen und generieren randomisiert neue Daten [9]. Synthetische Daten, also solche, die durch die trainierten generativen Modelle erzeugt werden, behalten idealerweise die statistischen Eigenschaften der Originaldaten bei, enthalten aber keine realen Informationen über die im Originaldatensatz abgebildeten Personen. Hierdurch besteht das Potenzial, dass eine bessere Abwägung zwischen Restrisiken und Datenvalidität erreicht werden kann als bei klassisch anonymisierten Daten. Eine Herausforderung besteht darin, dass im Regelfall große Menge an Trainingsdaten benötigt werden, um realistische synthetische Daten zu erzeugen.

### Erforschung sicherer Datennutzung im Projekt Künstliche Intelligenz am Forschungsdatenzentrum im BfArM zur Erforschung von Anonymisierungsmöglichkeiten und AI-readiness (KI-FDZ)

Das Projekt KI-FDZ zielt darauf ab, innovative Technologien zu untersuchen, die eine sichere Bereitstellung von Sekundärdaten für Forschungszwecke gewährleisten könnten, insbesondere in Anwendungsbereichen, die für das FDZ relevant sind. Ein besonderer Fokus liegt dabei auf KI, sowohl als Werkzeug für den Datenschutz als auch als Technologie zur Datenanalyse. Im Kontext dieses Artikels wird speziell auf Datenschutzaspekte eingegangen. Dazu zählen Maßnahmen wie Anonymisierung und KI-basierte Synthetisierung von Daten, die in KI-FDZ in Bezug auf den Erhalt der Validität der Daten und Restrisiken untersucht und verglichen werden. Das FDZ ist durch die Integration großer Datenmengen potenziell besonders gut für einen Einsatz von Synthetisierungsverfahren geeignet. Des Weiteren wird in KI-FDZ das Zusammenspiel von Anonymisierung oder Synthetisierung mit sicheren Verarbeitungsumgebungen untersucht, was potenziell zu einem besseren Gleichgewicht zwischen Datenschutz und Datennutzbarkeit beitragen könnte. Der Schwerpunkt von KI-FDZ im Bereich der sicheren Verarbeitungsumgebungen liegt in der Untersuchung, welchen Nutzen KI-Methoden für die Analyse und Modellierung von Abrechnungs- und Versorgungsdaten bieten und welche Werkzeuge den Nutzungsberechtigten zur Durchführung von KI-Experimenten in sicheren Umgebungen angeboten werden können.

## Erforschung von Methoden zur sicheren Datennutzung im Projekt KI-FDZ

### Grundkonzepte

Zur sicheren Sekundärnutzung von Gesundheitsdaten verschiedener Datenhalter wurde eine Reihe unterschiedlicher Verfahren vorgeschlagen, beispielsweise unter Nutzung von föderierten Datenanalysen oder Anonymisierung [10]. Bei allen bekannten Ansätzen ist es notwendig, für die Erreichung eines angemessenen Schutzniveaus Kompromisse auf der funktionalen Seite einzugehen (bspw. in Bezug auf die Datenvalidität oder die unterstützten Analysefunktionen). Das Projekt KI-FDZ untersucht einen Ansatz, bei dem Daten in einer sicheren Verarbeitungsumgebung bereitgestellt und dann mittels einer Kombination von Schutzmaßnahmen auf Daten- und Umgebungsebene in geschützter Form auswertbar gemacht werden.

**Figure 1:**
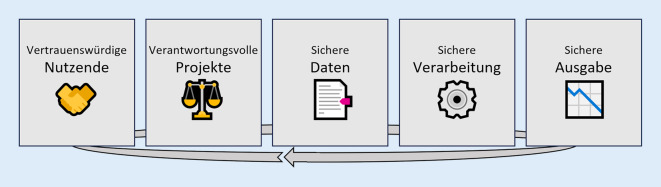

Das durch die Kombination von Maßnahmen erreichte Schutzniveau kann mittels des *Five Safes Framework* [11] bewertet werden. Wie in Abb. 1 dargestellt, strukturiert dieses Modell Maßnahmen anhand von 5 Achsen, die sich auf Datennutzende und Projekte sowie die Daten‑, Verarbeitungs- und Ergebnisebene beziehen. Die Kernidee ist, dass in Bezug auf die Vertrauenswürdigkeit der Nutzenden, die Qualität der Projekte sowie die Sicherheit auf Daten‑, Verarbeitungs- und Ausgabeebene Stellschrauben bestehen, die teilweise unabhängig voneinander justiert werden können, um optimalen Schutz bei optimaler Nutzbarkeit zu erreichen.

Wie bereits erwähnt, liegt der Schwerpunkt im Projekt auf Verfahren für sichere Daten und sichere Verarbeitungsumgebungen sowie deren Zusammenspiel. Bei klassischen Anonymisierungsverfahren werden personenbezogene Daten so modifiziert, dass die Personen, auf die sie sich beziehen, nicht mehr identifizierbar sind. Das kann beispielsweise durch Löschung, Generalisierung oder Aggregation von Einzelwerten erfolgen [12]. Bei der Datensynthetisierung werden – meist mittels KI-Verfahren – künstliche Datensätze erzeugt, die die statistischen Eigenschaften des Originaldatensatzes nachbilden, ohne auf realen individuellen Daten zu beruhen [13]. Während klassische Anonymisierungsverfahren also versuchen, die Identifizierung von Individuen zu verhindern, erzeugt die Datensynthese neue Daten, die keine direkte Beziehung zu den Originaldaten haben. Da sie aber versuchen, die statistischen Eigenschaften der Originaldaten nachzubilden, können auch Synthetisierungsverfahren potenziell Muster generieren, die Rückschlüsse auf die Daten von Einzelpersonen zulassen [14]. Sowohl für klassische Anonymisierungsverfahren als auch Synthetisierungsverfahren sollten deshalb Restrisikoanalysen durchgeführt werden. Aufgrund der Mehrschichtigkeit des untersuchten Lösungsansatzes können Restrisiken für die Privatheit der Betroffenen durch sichere Verarbeitungsumgebungen abgefangen werden. Auch kann ein mehrstufiges Verfahren umgesetzt werden, bei dem anonyme oder synthetische Daten für explorative Analysen und die Entwicklung von Algorithmen verwendet werden, die anschließend in einer sicheren Verarbeitungsumgebung auf den Echtdaten ausgeführt werden.

### Use Cases

Die Evaluation von Anonymisierungs- und Synthetisierungsverfahren hinsichtlich ihrer technischen Umsetzbarkeit und Risiko-Nutzen-Abwägung erfolgt im Projekt anhand zweier konkreter, exemplarischer Anwendungsbeispiele. Neben einer Bewertung von Restrisiken ermöglichen diese Use Cases auch die Evaluation der Validität geschützter Daten, indem Auswertungen auf den geschützten Daten und den Originaldaten ausgeführt und die Ergebnisse verglichen werden. Genutzt wird zum einen eine längsschnittliche Auswertung zur Untersuchung des Blutungsrisikos von Personen mit venöser Thromboembolie (VTE) unter oraler Antikoagulation und zeitgleicher Thrombozytenaggregationshemmer-Therapie (Infobox 1; [15]) und zum anderen eine querschnittliche populationsbasierte Auswertung zur Häufigkeit der Subtypen des Diabetes mellitus (Infobox 2). Beide Fragestellungen stellen häufige Anwendungsfälle von GKV-Routinedaten dar und bilden damit realistische Anforderungen an zukünftige Nutzungsanfragen an das FDZ ab.

Grundlage für die in den Use Cases durchgeführten Analysen ist die Forschungsdatenbank der InGef, die Abrechnungsdaten von 51 gesetzlichen Krankenversicherungen enthält. Ausschließlich Mitarbeitende der InGef haben lesenden Zugriff auf die Daten der Forschungsdatenbank, die nur in aggregierter Form ausgegeben werden. Die Analysen folgen den Empfehlungen der Guten Epidemiologischen Praxis (GEP; [16]) sowie der Guten Praxis Sekundärdatenanalyse (GPS; [17]).

Für die Auswertungen werden Daten von etwa 8 Mio. Versicherten der Jahre 2014 bis 2019 genutzt. Neben soziodemografischen Informationen enthalten die Forschungsdaten der InGef Informationen über ambulante Leistungen und Diagnosen, Krankenhausdaten einschließlich der Aufnahmezeiten, der Haupt- und Nebendiagnosen und der durchgeführten Operationen und Prozeduren, Daten über die Verschreibung von Medikamenten, Informationen über Hilfsmittel und Heilmittel sowie über die Kosten, die in diesen Bereichen angefallen sind [18]. Damit weisen diese Forschungsdaten große Parallelen mit den Daten des Forschungsdatenzentrums auf.

Die auf Basis der Use Cases generierten anonymisierten und synthetisierten Datensätze werden analog der Originaldatensätze, d. h. mit den gleichen Auswertungsskripten, analysiert. Dies ermöglicht eine anschließende Bewertung der Nützlichkeit bzw. Validität der anonymisierten und synthetisierten Ausgabedaten. Die Bewertung erfolgt für Use Case 1 zunächst über einen direkten Vergleich der Verteilung der betrachteten Kovariaten und den für die *Propensity-Score*-Berechnung ausgewählten Kovariaten. Anschließend werden die berechneten Hazard Ratios zwischen den Datensätzen verglichen, indem als Nutzenmaße die Überlappung der Konfidenzintervalle betrachtet wird [19, 20]. Angepasst an den verwendeten Datensatz werden zur Bewertung der Nützlichkeit für Use Case 2 andere geeignete Metriken eingesetzt. So ist die Überlappung der Konfidenzintervalle für große Stichproben nur bedingt aussagekräftig. Neben dem Vergleich der Verteilung und der Ähnlichkeit der Variablen werden absolute und relative Differenzen, *Standardized Mean Difference* (SMD) und *Ratio of Estimates* (ROE) bestimmt [20, 21].

### Geschützte Daten: Anonymisierung und Synthetisierung

Um die Verwendung von Anonymisierungs- und Synthetisierungsverfahren vorzubereiten, Restrisiken zu quantifizieren und Verfahren auf einen kombinierten Einsatz in sicheren Verarbeitungsumgebungen abzustimmen, werden Bedrohungsszenarien modelliert, die potenzielle Risiken für die Privatheit beschreiben. Dafür wurde im Projekt KI-FDZ ein Verfahren spezifiziert, bei dem zunächst Attribute mit erhöhtem Reidentifizierungsrisiko identifiziert werden. Direkt identifizierende Merkmale, wie z. B. Namen oder Versicherungsnummern, werden grundsätzlich nicht für Forschungszwecke zur Verfügung gestellt. Diese können beispielsweise durch Abgleich mit Listen von besonders risikobehafteten Merkmalen in Gesetzen und Leitfäden identifiziert werden (vgl. bspw. [22, 23]). Datenübermittlungen an das FDZ finden in pseudonymisierter Form statt, weshalb nur Pseudonyme als direkt identifizierende Merkmale relevant sind. In einem weiteren Schritt werden „Schlüsselvariablen“ identifiziert, die beispielsweise in Kombination eine Verknüpfung mit anderen Datensätzen oder mit Hintergrundwissen der Nutzenden ermöglichen können. Dazu werden Variablen anhand ihrer Stabilität, Verfügbarkeit und Unterscheidbarkeit klassifiziert [24]. Für diese Prozessschritte wurde eine Vorlage entwickelt, die als Werkzeug die Risikobewertung unterstützt und statistische Informationen über die Eigenschaften der Variablen im Datensatz darstellt. Diese methodische Herangehensweise ermöglicht es, unterschiedliche Bedrohungsszenarien zu modellieren, und bildet die Grundlage für die anschließende Anonymisierung der Datensätze.

Aufbauend auf den Ergebnissen der Risikoanalyse werden Anonymisierungsverfahren eingesetzt, um die identifizierten Risiken zu reduzieren. Die eingesetzten Verfahren fokussieren dabei auf die von der Artikel-29-Datenschutzgruppe als besonders relevant identifizierten Risiken [25]: (1) *Aussondern* bezieht sich auf die Möglichkeit, ein Individuum in einem Datensatz zu identifizieren und es von anderen zu unterscheiden, (2) *Verknüpfbarkeit* beschreibt die Möglichkeit, 2 Datensätze, die dasselbe Individuum betreffen, zu verknüpfen, (3) *Inferenz* ist ein Vorgang, bei dem Informationen über ein Individuum aus anderen verfügbaren Daten abgeleitet werden können, auch ohne die Daten des Individuums direkt zu identifizieren.

Zur Anonymisierung der Datensätze wird in KI-FDZ eine Reihe von Verfahren untersucht. Dazu gehören insbesondere Methoden, die die Eindeutigkeit der in der Bedrohungsanalyse als schützenswert identifizierten Merkmalskombinationen reduzieren [26], was Aussondern und Verknüpfung verhindert. Dazu kommen solche, die sicherstellen sollen, dass die Informationen, die über einzelne Personen abgeleitet werden können, nicht viel präziser sind als die Informationen, die aus der Gesamtmenge aller Daten über alle Personen abgeleitet werden können [27], was Inferenz adressiert. Neben den Eigenschaften der für Forschungszwecke relevanten Sekundärdaten selbst, können auch Informationen über die zugrunde liegende Gesamtpopulation berücksichtigt werden [28]. Zur Reduktion der Risiken können gezielt Veränderungen an den Daten durchgeführt werden [12]. Untersucht werden im Projekt KI-FDZ unter anderem Verfahren zur Vergröberung von Attributwerten (beispielsweise kann das genaue Alter einer Person durch eine Altersgruppe ersetzt werden), Verfahren zur Aggregation von numerischen Werten in einen gemeinsamen Wert durch Clusterbildung sowie das Löschen von Datensätzen, Variablen oder Einzelwerten. Zur praktischen Umsetzung können verschiedene Softwarelösungen eingesetzt werden [29].

Moderne Synthetisierungsverfahren verwenden eine ganze Bandbreite an methodischen Ansätzen mit einem besonderen Fokus auf künstliche neuronale Netzwerke und Deep-Learning-Verfahren. Eine große Zahl an Synthetisierungsverfahren basiert auf *Generative Adversarial Networks* (GANs; [30]). Diese sind eine Art von KI-Verfahren, die darauf ausgelegt sind, neue Daten zu generieren, die ähnlichen Mustern wie die ursprünglichen Trainingsdaten folgen. Dazu werden 2 neuronale Netzwerke gemeinsam trainiert: ein Generatornetzwerk, das synthetische Daten generiert, und ein Diskriminatornetzwerk, das versucht, generierte und Originaldaten zu unterscheiden. Andere verwendete Ansätze umfassen unter anderem *Bayesian Networks, Variational Autoencoders* oder Transformer-Modelle.

**Figure 2:**
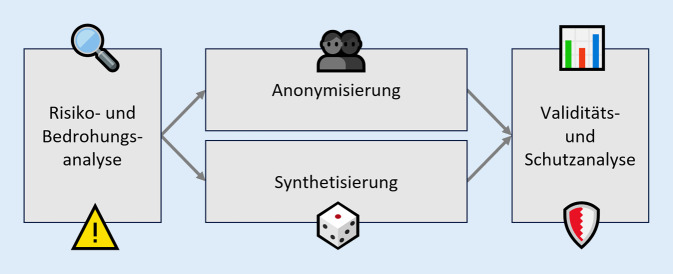

Während Bayesian Networks wie PrivBayes [31] die Verteilungen und Korrelationen in den Daten mittels einer Netzwerkstruktur modellieren, sind Variational Autoencoders wie VAEM [32] KI-Verfahren, bei denen ähnlich zu den GANs 2 neuronale Netzwerke kombiniert werden: ein Encoder-Netzwerk und ein Decoder-Netzwerk, die zusammen versuchen die Verteilung der Originaldaten in einem kompakten Format zu lernen und zu generalisieren. Transformer-Modelle sind vor allem durch große Sprachmodelle (Large Language Models, LLMs) wie ChatGPT bekannt geworden. REaLTabFormer [33] verwendet eine spezielle Vorverarbeitung der Daten, um vortrainierte Transformer-Modelle wie GPT‑2 auch für die Synthetisierung von tabellarischen Daten nutzbar zu machen. Spezielle GAN-Verfahren für medizinische Anwendungen umfassen medGAN [34], das eine Kombination aus einem Autoencoder und einem GAN nutzt, um hochdimensionale synthetische Patientendaten mit diskreten Variablen zu generieren. *Variational Autoencoder Modular Bayesian Networks* (VAMBN; [35]) kombiniert Autoencoder und Bayesian Network und kann bei der Erzeugung von Patiententrajektorien mit einer hohen Anzahl an Variablen, heterogenen Datentypen und fehlenden Werten umgehen. Die Qualität der erzeugten Daten kann je nach verwendetem Algorithmus und Datensatz deutlich variieren [9, 36], weshalb eine Überprüfung der Validität und des erreichten Schutzniveaus der synthetischen Daten sehr wichtig ist (Abb. 2).

Die Implementierung von klassischen Anonymisierungsverfahren und KI-basierten Synthetisierungsmethoden im Rahmen von KI-FDZ erlaubt nicht nur eine ganzheitliche Betrachtung von Schutzmaßnahmen auf Datenebene, sondern ermöglicht auch einen Vergleich der untersuchten Anonymisierungs- und Synthetisierungsverfahren in Bezug auf den erreichten Schutz und die Validität der Ausgabedaten (siehe Abschnitt „Evaluation und Zusammenspiel der Schutzmaßnahmen“ unten). Das Zusammenspiel der einzelnen Schritte ist in Abb. 2 dargestellt.

### Geschützter Zugang: Sichere Verarbeitungsumgebungen

Neben Maßnahmen auf Datenebene können auch auf der Ebene der Verarbeitungen wirksame Maßnahmen zum Datenschutz getroffen werden. Ein wichtiger grundlegender Schritt liegt dabei darin, dass aufbauend auf Nutzungsanträgen und Authentifizierungs- sowie Autorisierungsfunktionen projektspezifische Datensätze in sicheren Verarbeitungsumgebungen bereitgestellt werden. Nutzungsberechtigte definieren im Rahmen des Antragsprozesses, welche Variablen sie für ihre Auswertung nutzen möchten. Nachdem der Antrag erfolgreich geprüft wurde, wird den Nutzenden eine sichere Umgebung zugewiesen, in der die Berechnungen durchgeführt werden können. Dies ermöglicht auch eine Abstimmung der Zugänglichkeit von Verarbeitungen und deren Ergebnissen auf Zugriffsberechtigungen und Vertrauensstufen von Nutzenden. Außerdem erlaubt die Container-Technologie die Kapselung von Rechenprozessen in abgeschotteten Umgebungen, den sogenannten Containern, was eine Isolation verschiedener Verarbeitungsumgebungen und damit eine Erhöhung des Schutzes bewirkt [37].

Innerhalb der Verarbeitungsumgebungen können umfangreiche Analysemöglichkeiten, bspw. mittels statistischer Verfahren und Data-Science-Methoden, angeboten werden. Im Gegensatz zur Herausgabe von Daten an Nutzende besteht aber die Möglichkeit, die verwendbaren Verfahren zu kontrollieren und das Verhalten der Nutzenden im Analyseraum zu monitoren. Im Projekt KI-FDZ werden unterschiedliche Verarbeitungsumgebungen berücksichtigt, die modulare Funktionen von Statistik- über Visualisierungs- bis zu Machine-Learning-Methoden bereitstellen können und so eine hohe Flexibilität bzgl. unterschiedlicher Anwendungsfälle bieten.

KI-Lernverfahren sind langläufig, wobei einzelne Teilschritte besonders gut gekapselt werden können. In KI-FDZ wird eine sogenannte AI-Sandbox implementiert, die die testweise Erstellung und Erprobung von Machine-Learning-Pipelines auf Gesundheitsdaten erlaubt. Das erarbeitete Konzept sieht vor, KI-Algorithmen aus bestehenden Paketen in den Sprachen Python (z. B. die Open-Source-Software scikit-learn, TensorFlow, PyTorch) und R (z. B. die Open-Source-Software mlr3) anzubieten. Die Algorithmen sollen über ein ansprechendes Nutzerinterface auswählbar und kombinierbar sein. Neben der Algorithmenzusammenstellung und Ergebnisansicht sind eine Komponente zur Visualisierung von Datenverteilungen sowie eine Beratungskomponente geplant, die auf relevante Publikationen verweist und die Nutzenden bei der Zusammenstellung von KI-Pipelines unterstützen kann.

Im Gegensatz zu statistischen Analysen, deren Ergebnisse sich in tabellarischer Form relativ einfach aggregiert zusammenfassen lassen, sind die Ergebnisse von KI-Algorithmen komplexer. Die Frage der Ergebnisbereitstellung und der sich daraus ergebenden datenschutzrelevanten Konsequenzen wird ebenfalls in KI-FDZ untersucht. Aus der Perspektive der Forschenden sollte es möglich sein, die gewählte Algorithmik, eingehende Daten sowie die auf unabhängigen Validierungsdaten erzielte Modellgüte benennen zu können, sodass gewonnene Erkenntnisse ihren Weg in die Anwendung und wissenschaftliche Diskussion finden können.

### Evaluation und Zusammenspiel der Schutzmaßnahmen

**Figure 3:**
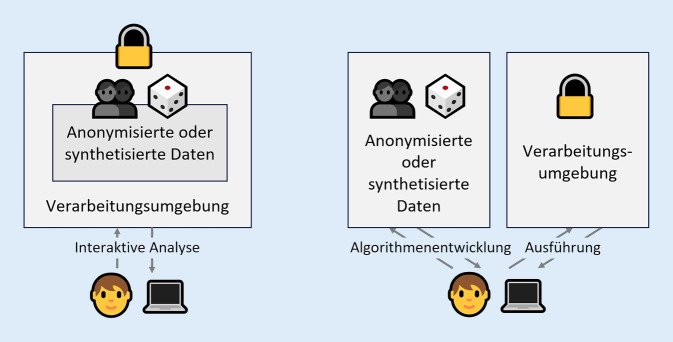

Der in KI-FDZ untersuchte Prozess sieht vor, dass sich Anonymisierung, Synthetisierung und sichere Verarbeitungsumgebungen ergänzen und so einen mehrschichtigen Ansatz zum Schutz der Privatheit umsetzen. Für die Kombination der Verfahren gibt es unterschiedliche Optionen, die auf die spezifischen Anforderungen der Nutzenden abgestimmt werden können. Ein Überblick über 2 mögliche Szenarien ist in Abb. 3 dargestellt.

In beiden Szenarien werden der Zugang zu den Daten und deren Verarbeitung kontrolliert. Die Rollen von Anonymisierungs- bzw. Synthetisierungsmaßnahmen und der sicheren Verarbeitungsumgebung sind aber unterschiedlich. Im *interaktiven Szenario* wird den Nutzenden ein direkter Zugang zur Auswertung von Daten innerhalb einer Umgebung angeboten. Dabei schränkt die Verarbeitungsumgebung die Interaktionsmöglichkeiten mit den Daten so ein, dass bspw. (unautorisierte) Verknüpfungen mit anderen Datenquellen verhindert werden. Anonymisierungs- oder Synthetisierungsprozesse können deshalb so gestaltet sein, dass sie vor allem „unbeabsichtigtes Erkennen“ von Personen verhindern, was es wiederum erlaubt, die Daten auf Validität zu optimieren [38]. Im *nicht-interaktiven Szenario *werden starke Anonymisierungs- oder Synthetisierungsmethoden eingesetzt, um Daten ohne Personenbezug zu erzeugen. Da dies unter Umständen bedeutet, dass Abstriche bei der Validität gemacht werden müssen, werden diese Daten Nutzenden ausschließlich für die Algorithmenentwicklung zur Verfügung gestellt. Die Ausführung der entwickelten Analysen findet anschließend vollständig abgeschottet in einer sicheren Verarbeitungsumgebung auf den Originaldaten statt. Eine integrierte Betrachtung des erreichten Schutzniveaus kann auf qualitativer Ebene u. a. mittels des LINDDUN^1^-Ansatzes und des Five-Safes-Modells durchgeführt werden [39]. Auf quantitativer Ebene ist es beispielsweise möglich, die aus den Daten abgeleiteten Wahrscheinlichkeiten für erfolgreiche Reidentifizierungen unter Berücksichtigung der durch sichere Verarbeitungsumgebungen verringerten Wahrscheinlichkeiten für Reidentifizierungsversuche zu betrachten.

Da Maßnahmen auf Datenebene, insbesondere die Anonymisierung oder Synthetisierung, immer eine Balance zwischen der Validität der Ausgabedaten und dem erzielten Schutzniveau erfordern, werden beide Aspekte im Projekt gründlich evaluiert. Eine gebräuchliche Methode zur robusten Evaluation der Validität von geschützten Daten besteht darin, Analysen sowohl auf den Originaldaten als auch auf den geschützten Daten durchzuführen und die Reproduzierbarkeit sowie mögliche Verzerrungen zu betrachten. Dies wird in KI-FDZ mithilfe der Use Cases (siehe oben) umgesetzt.

**Figure 4:**
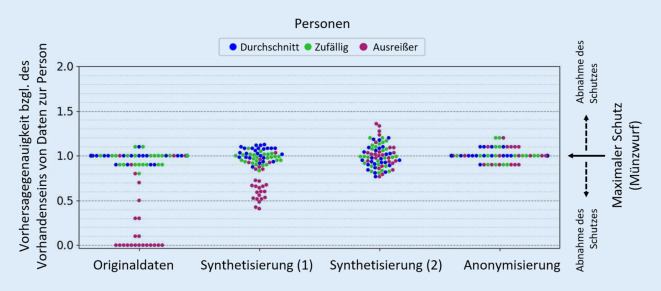

Zur Evaluierung des erzielten Schutzniveaus gibt es verschiedene Ansätze, die meist für spezifische Anonymisierungsverfahren entwickelt wurden [40]. Ein Hauptziel im Projekt ist die Entwicklung generischer Verfahren, die auf eine Vielzahl von Anonymisierungs- und Synthetisierungsverfahren angewandt werden können und so eine Vergleichbarkeit der Ergebnisse von Restrisikoanalysen bieten. Besonders im Fokus stehen KI-basierte Verfahren, bei denen Modelle so trainiert werden, dass sie das Vorhandensein oder Fehlen von Informationen zu einer spezifischen Person in geschützten Datensätzen erkennen können, was eine spezifische Form der Inferenz (siehe Abschnitt „Geschützte Daten: Anonymisierung und Synthetisierung“) darstellt [14]. Ein exemplarisches Ergebnis solcher Verfahren ist in Abb. 4 dargestellt. Sie zeigt für einen Beispieldatensatz und 3 durch Synthetisierung oder Anonymisierung gewonnene geschützte Versionen dieses Datensatzes sowie eine Auswahl von Personen, wie gut eine solche Vorhersage gelingt. Ein Wert von 1.0 auf der y‑Achse symbolisiert einen vollständigen Schutz (Vorhersagegenauigkeit entspricht einem Münzwurf), wohingegen Abweichungen nach oben oder unten auf eine prädiktive Leistung des Modells hindeuten. Details zum Verfahren sind in [14] beschrieben. Es ist zu sehen, dass selbst im unveränderten Originaldatensatz zufällig ausgewählte Personen und Personen mit durchschnittlichen Merkmalen gut geschützt sind, während Personen mit besonders einzigartigen Werteausprägungen (Ausreißer) leichter erkannt werden können. Traditionelle Anonymisierungsverfahren sowie KI-basierte Synthetisierungsverfahren bieten einen messbaren Schutz vor Vorhersagen bezüglich des Vorhandenseins von Daten spezifischer Personen. Wir weisen darauf hin, dass diese Grafik nicht geeignet ist, um die Überlegenheit oder Unterlegenheit einzelner Verfahren abzuleiten, da nur ein einfacher Beispieldatensatz genutzt wurde und für eine abschließende Bewertung insbesondere die erzielte Abwägung zwischen Schutz und Validität der Daten berücksichtigt werden muss. Die vorläufigen Ergebnisse der Evaluationen der Use Cases von KI-FDZ legen den Schluss nahe, dass synthetische Daten eine beträchtliche Validität für komplexe Analysen aufweisen können. Dies deutet auf eine günstige Risiko-Nutzen-Relation hin. Weitere Untersuchungen sind jedoch notwendig, um diese Erkenntnisse zu erhärten und zu verallgemeinern. Die abschließende Bewertung der Anonymisierungsverfahren und der Vergleich der untersuchten Anonymisierungs- und Synthetisierungsverfahren ist ebenfalls noch nicht abgeschlossen.

## Fazit

Das Projekt KI-FDZ untersucht einen mehrschichtigen Datenschutzansatz, der auf einer abgestimmten Kombination von Anonymisierungs- und Synthetisierungsmaßnahmen mit sicheren Verarbeitungsumgebungen beruht. Dieses Modell verfolgt das Ziel, einen robusten und dennoch flexiblen Schutzmechanismus zu schaffen, der sowohl die Privatheit der Betroffenen als auch die Nutzbarkeit der Daten gewährleistet.

Die bisher im Projekt erreichten Ergebnisse legen nahe, dass ein hohes Maß an Datenschutz und gleichzeitig eine hohe Datenvalidität erreichbar sind. Diese Erkenntnisse unterstreichen das enorme Potenzial von KI-Methoden zur Verbesserung sowohl der Verfügbarkeit von Daten als auch des Datenschutzes. Mit dem richtigen Einsatz von KI-Verfahren kann die Sekundärnutzung von Gesundheitsdaten potenziell signifikant verbessert werden, ohne dabei Kompromisse beim Datenschutz eingehen zu müssen. Der im Projekt untersuchte Ansatz kann einen wichtigen Baustein bei der sicheren Sekundärnutzung von Gesundheitsdaten darstellen.

Die Forschung im Bereich sicherer Verarbeitungsumgebungen hat in den vergangenen Jahren aufgrund der zentralen Rolle im geplanten Europäischen Gesundheitsdatenraum (EHDS) stark zugenommen. Wichtige Projekte rund um den EHDS sind u. a. TEHDAS [41], die Joint Action Towards the European Health Data Space, und der EHDS2 Pilot [42] sowie weitere Projekte, die von der Konzeptentwicklung [43] bis zur Implementierung für konkrete Anwendungsbereiche [44] reichen. Die in KI-FDZ untersuchten Verfahren werden auch in einigen dieser Projekte durch die Partner eingebracht.

### Infobox 1 Use Case 1 – Venöse Thromboembolie

**Fragestellung**: Wirksamkeit und Sicherheit der gleichzeitigen Verwendung von direkten oralen Antikoagulanzien (DOAKs) oder Vitamin-K-Antagonisten (VKA) und Thrombozytenaggregationshemmern bei Patientinnen und Patienten mit venöser Thromboembolie (VTE; [15]).

**Studiendesign:** retrospektive Kohortenstudie; Patientinnen und Patienten mit inzidenter VTE in den Jahren 2014–2019.

**Analysedatensatz: **geringe Komplexität bzgl. Synthetisierung/Anonymisierung (eine Zeile pro Versicherten; 61 Variablen).

**Analysemethode**: Bestimmung der Inzidenzrate der Endpunkte – schwere Blutungen, Gesamtmortalität und erneute VTE; Bewertung der Ergebnisse durch Berechnung von Hazard Ratios mittels Cox Proportional Hazards Models; Propensity Score; Inverse Probability-of-Treatment-Weighting-Schätzung.

### Infobox 2 Use Case 2 – Diabetes mellitus

**Fragestellung**: Beschreibung der Epidemiologie des Diabetes mellitus sowie der demografischen und klinischen Merkmale der Patientinnen und Patienten.

**Studiendesign:** querschnittliche, retrospektive Kohortenstudie; Versicherte der Jahre 2018 und 2019.

**Analysedatensatz:** mittlere Komplexität bzgl. Synthetisierung/Anonymisierung (Zeitreihenanalyse; 5 Zeilen pro Versicherten; 67 Variablen).

**Analysemethode**: deskriptive Statistik von Komorbiditäten, Verschreibungen und Demografie nach Diabetes-Typ.

## Footnotes

1 LINDDUN: Linkability, Identifiability, Non-repudiation, Detectability, Disclosure of Information, Unawareness, Non-compliance.

## References

[1] Kreis K, Neubauer S, Klora M, Lange A, Zeidler J (2016). Status and perspectives of claims data analyses in Germany—a systematic review. Health Policy.

[2] Slagman A, Hoffmann F, Horenkamp-Sonntag D, Swart E, Vogt V, Herrmann WJ (2023). Analyse von Routinedaten in der Gesundheitsforschung: Validität, Generalisierbarkeit und Herausforderungen. Z Allg.

[3] Neubauer S, Kreis K, Klora M, Zeidler J (2017). Access, use, and challenges of claims data analyses in Germany. Eur J Health Econ.

[4] FDZ Gesundheit. https://www.forschungsdatenzentrum-gesundheit.de. Zugegriffen: 1. Okt. 2023

[5] Bundesdatenschutzgesetz vom 30. Juni 2017 (BGBl. I S. 2097), das zuletzt durch Artikel 10 des Gesetzes vom 23. Juni 2021 (BGBl. I S. 1858; 2022 I 1045) geändert worden ist 2021.

[6] Verordnung (EU) 2016/679 des Europäischen Parlaments und des Rates vom 27. April 2016 zum Schutz natürlicher Personen bei der Verarbeitung personenbezogener Daten, zum freien Datenverkehr und zur Aufhebung der Richtlinie 95/46/EG (Datenschutz-Grundverordnung).

[7] Aggarwal CC (2005). On k-anonymity and the curse of dimensionality. VLDB Endowment.

[8] Chen RJ, Lu MY, Chen TY, Williamson DFK, Mahmood F (2021). Synthetic data in machine learning for medicine and healthcare. Nat Biomed Eng.

[9] Goncalves A, Ray P, Soper B, Stevens J, Coyle L, Sales AP (2020). Generation and evaluation of synthetic patient data. BMC Med Res Methodol.

[10] Wirth FN, Meurers T, Johns M, Prasser F (2021). Privacy-preserving data sharing infrastructures for medical research: systematization and comparison. BMC Med Inform Decis Mak.

[11] Desai T, Ritchie F, Welpton R (2016). Five safes: designing data access for research. Econ Work Pap Ser.

[12] El Emam K (2013). Guide to the de-identification of personal health information.

[13] Bellovin SM, Dutta PK, Reitinger N (2018). Privacy and synthetic datasets. SSRN Electron J.

[14] Stadler T, Oprisanu B, Troncoso C (2020) Synthetic data—anonymisation groundhog day. 10.48550/ARXIV.2011.07018

[15] Douros A, Basedow F, Cui Y, Walker J, Enders D, Tagalakis V (2022). Effectiveness and safety of direct oral anticoagulants with antiplatelet agents in patients with venous thromboembolism: a multi-database cohort study. Res Pract Thromb Haemost.

[16] Hoffmann W, Latza U, Baumeister SE, Brünger M, Buttmann-Schweiger N, Hardt J, Hoffmann V, Karch A, Richter A, Schmidt CO, Schmidtmann I, Swart E, van den Berg N (2019). Guidelines and recommendations for ensuring Good Epidemiological Practice (GEP): a guideline developed by the German society for epidemiology. Eur J Epidemiol.

[17] Swart E, Gothe H, Geyer S, Jaunzeme J, Maier B, Grobe TG, Ihle P (2015). Gute Praxis Sekundärdatenanalyse (GPS): Leitlinien und Empfehlungen. Gesundheitswesen.

[18] Ludwig M, Enders D, Basedow F, Walker J, Jacob J (2022). Sampling strategy, characteristics and representativeness of the InGef research database. Public Health.

[19] Karr AF, Kohnen CN, Oganyan A, Reiter JP, Sanil AP (2006). A framework for evaluating the utility of data altered to protect confidentiality. Am Stat.

[20] Snoke J, Raab GM, Nowok B, Dibben C, Slavkovic A (2018). General and specific utility measures for synthetic data. J R Stat Soc Ser A Stat Soc.

[21] Taub J, Elliot M, Sakshaug JW (2020). The impact of synthetic data generation on data utility with application to the 1991 UK samples of anonymised records. Trans Data Priv.

[22] Office for Civil Rights (2002). Standards for privacy of individually identifiable health information. Final rule. Fed Regist.

[23] European Medicines Agency (2018). External guidance on the implementation of the European Medicines Agency policy on the publication of clinical data for medicinal products for human use.

[24] Malin B, Loukides G, Benitez K, Clayton EW (2011). Identifiability in biobanks: models, measures, and mitigation strategies. Hum Genet.

[25] Article 29 Data Protection Working Party (2014) Opinion 05/2014 on Anonymisation Techniques. 0829/14/EN WP216

[26] Sweeney L (2002). k-anonymity: a model for protecting privacy. Int J Uncertain Fuzziness Knowledge-based Syst.

[27] Li N, Li T, Venkatasubramanian S (2007). t-Closeness: Privacy Beyond k-Anonymity and l-Diversity. Proc Int Conf Data Eng.

[28] Hoshino N (2001). Applying pittman’s sampling formula to microdata disclosure risk assessment. J Off Stat.

[29] Haber AC, Sax U, Prasser F, NFDIHealth Consortium (2022). Open tools for quantitative anonymization of tabular phenotype data: literature review. Brief Bioinform.

[30] Hernandez M, Epelde G, Alberdi A, Cilla R, Rankin D (2022). Synthetic data generation for tabular health records: a systematic review. Neurocomputing.

[31] Zhang J, Cormode G, Procopiuc C, Srivastava D, Xiao X (2014). PrivBayes: Private data release via Bayesian networks. Proc ACM SIGMOD Int Conf Manag Data.

[32] Ma C, Tschiatschek S, Hernández-Lobato JM, Turner R, Zhang C (2020). VAEM: a Deep Generative Model for Heterogeneous Mixed Type Data.

[33] Solatorio AV, Dupriez O (2023). REaLTabFormer: Generating Realistic Relational and Tabular Data using Transformers.

[34] Choi E, Biswal S, Malin B, Duke J, Stewart WF, Sun J (2018). Generating Multi-label Discrete Patient Records using Generative Adversarial Networks.

[35] Gootjes-Dreesbach L, Sood M, Sahay A, Hofmann-Apitius M, Fröhlich H (2019). Variational Autoencoder Modular Bayesian Networks (VAMBN) for Simulation of Heterogeneous Clinical Study Data. Bioinformatics.

[36] Tao Y, McKenna R, Hay M, Machanavajjhala A, Miklau G (2022). Benchmarking Differentially Private Synthetic Data Generation Algorithms.

[37] Docker Security—OWASP Cheat Sheet Series. https://cheatsheetseries.owasp.org/cheatsheets/Docker_Security_Cheat_Sheet.html. Zugegriffen: 1. Okt. 2023

[38] Prasser F, Kohlmayer F, Spengler H, Kuhn KA (2018). A scalable and pragmatic method for the safe sharing of high-quality health data. IEEE J Biomed Health Inform.

[39] Deng M, Wuyts K, Scandariato R, Preneel B, Joosen W (2011). A privacy threat analysis framework: supporting the elicitation and fulfillment of privacy requirements. Requir Eng.

[40] Wagner I, Eckhoff D (2018). Technical privacy metrics: a systematic survey. ACM Comput Surv.

[41] Joint Action Towards the European Health Data Space—TEHDAS. https://tehdas.eu/. Zugegriffen: 1. Okt. 2023

[42] Home—EHDS2 Pilot—Official website. https://ehds2pilot.eu/. Zugegriffen: 1. Okt. 2023

[43] Home. HealthyCloud. https://healthycloud.eu/. Zugegriffen: 1. Okt. 2023

[44] Stamm T, Bott N, Thwaites R, Mosor E, Andrews MR, Borgdorff J et al (2021) Building a value-based care infrastructure in Europe: the health outcomes observatory. NEJM Catal Innov Care Deliv 2:

